# Very fast evolution, not-so-fast publication—a proposed solution

**DOI:** 10.1093/nsr/nwaa010

**Published:** 2020-01-29

**Authors:** Chung-I Wu, Mu-ming Poo

**Affiliations:** 1 Section Editor for Life Sciences at NSR; 2 Executive Editor-in-Chief of NSR

In this editorial, we make the following points: The main threat of the new coronavirus (2019-nCoV, now officially named as SARS-CoV-2) may be its continual evolution in human populations, a lesson from SARS of 2002–2003. Extensive genomic sequencing will identify the highly infectious strains. Hence, such sequences must be collected and disseminated in real time. Academic journals like NSR can facilitate the rapid dissemination.

The new coronavirus identified from a cluster of pneumonia cases in Wuhan, China, has become a widespread public-health concern. The timing of the outbreak is particularly unfortunate as it coincides with the peak of a virulent season of flu, which has caused thousands of death worldwide. A case of flu in China, which should be rather common, now comes with a degree of anxiety or panic, exerting further strains on the medical system.

Several publications have appeared in a short period of time [1–7]. This new 2019-nCoV virus appears to originate in bats. Indeed, Zhou *et al*. [7] has identified a bat coronavirus that has a 96.2% similarity to the 2019-nCoV. While a secondary animal host seems plausible from studies of other coronaviruses, its identity remains elusive. The 2019-nCoV has now been classified as a distant relative of the SARS virus ([1,7,8]; see Fig. 1 in page 240 in this issue).

## THE THREAT – RAPID VIRAL EVOLUTION IN HUMAN POPULATIONS

Public concern of the spread of viruses is rightly about ‘quantity’ – the more viruses infecting people will beget more infections. However, there is an even more worrisome qualitative change that is driven by natural selection. Increasingly infectious strains will emerge, first as minor clones that carry mutations for a proliferative advantage. Subsequently, the minor clones will increase in number to become dominant ones, thus amplifying the opportunities for further evolution. Evolution does not stop after viruses leaving the bats as the real action may happen in human populations.

Such evolution in infectiousness within human populations was documented in the spread of the SARS-CoVs in 2002–2003 when the super-infectious strain gradually evolved. This evolution can be gleaned from the genomes of the viral strains. For SARS, the most aggressive strain had multiple sequence changes in key genes, which took time to evolve. In principle, one expects the gradual emergence of dominant clones that are found in a larger and larger fraction of patients. A dominant clone may eventually evolve into a super-infectious strain. No less important, minor clones that have a long phylogenetic branch (i.e., many mutations) could be poised to become dominant in the days to come.

## THE THREAT OF 2019-nCoV AS OF MID-JANUARY

At present, the published analyses of 2019-nCoVs focus on the divergence from their relatives in bats[1,4,7]. These published sequences are too few to elucidate the viral evolution in humans. Nevertheless, the 30 genomic sequences available to the public do offer a quick glimpse (http://virological.org/; https://www.gisaid.org). As discussed in Wei *et al*. [8], the evolution of 2019-nCoVs in humans had been modest in the first half of January of 2020. The good news is that, during this period, various strains have been evolving in parallel without any one dominating the rest.

An exception may be a small cluster of four samples, three from Guangdong and one from US. One might wonder whether these 4 samples are the harbinger of something bigger. The question is then how much more evolution may have happened between mid-January and now. The up-tick of new cases since mid-January could be an indication of further evolution in 2019-nCoVs. These are questions to be answered by further work.

## FAST EVOLUTION VS SLOW PUBLICATION – A PROPOSED RESOLUTION

It is clear that the sequencing of 2019-nCoVs has to be expanded to include samples from all geographical areas and in continual time. The sequences will reveal the most threatening strains and their identification may inform treatment decisions and drug development. The quarantine practice may also use such information, which should be available to the research community and the public at the earliest time possible.

Unfortunately, it looks increasingly likely that research teams may withhold the information until a formal publication is accepted. Controversies surrounding the unauthorized use of unpublished genomic sequences have heated up on the internet. The speed of academic publishing, however, may not meet this demand for the real-time release of sequence data. For example, while the publications of references [1–3] are almost ultrasonic in speed for science publishing, the sequences are already dated at the time of formal publication. The conclusions have already been known in earlier controversial papers[4–7].

We now propose that journals start to publish these sequences as formal publications that should have adequate descriptions of the samples and full sequences. Clearly, the analyses and interpretations would be light but clinically important information can often be obtained by such preliminary analyses. By doing so, we assure the authors of the sequencing work be properly credited with a citable publication. They and others can then proceed with the more rigorous analyses and interpretations that will benefit the general public. NSR will accept such submissions for quick review by the editorial board.

## THE FOLLOW-UP

After the current crisis is under control, it will be necessary for China to address the root problems of the wildlife commerce. Societies that have dense populations, which are particularly susceptible to contagions, tend to be isolated from wild lives. Human settlements in frequent contact with wild lives also tend to have low densities. High-density human populations and wild animals are a dangerous mix, which should be stringently regulated, if not eliminated. This current episode should be a second lesson, less than 20 years after SARS.

## References

[1] Zhu N , Zhang DY, Wang WL et al. Int J Infect Dis 2020; 382: 727–33.

[2] Chan JF , Yuan SF, Kok KH et al. Lancet 2020; 395: 514–52. 31986261 10.1016/S0140-6736(20)30154-9 PMC7159286

[3] Huang CL , Wang YM, Li XW et al. Lancet 2020; 395: 497–506. 31986264 10.1016/S0140-6736(20)30183-5 PMC7159299

[4] Xu XT , Chen P, Wang JF et al. Sci China Life Sci 2020; 63: 457–60. 32009228 10.1007/s11427-020-1637-5 PMC7089049

[5] Lu H , Stratton CW, Tang YW. J Med Virol 2020; doi:10.1002/jmv.25678.

[6] Hui SD , I Azhar E, Madani TA et al. Int J Infect Dis 2020; 91: 264–6. 31953166 10.1016/j.ijid.2020.01.009 PMC7128332

[7] Zhou P , Yang XL, Wang XG et al. bioRxiv 2020; 10.1101/2020.01.22.914952.

[8] Wei X , Li X, Cui J. Natl Sci Rev 2020; 7: 239–42. 32288962 10.1093/nsr/nwaa009 PMC7107983

